# Evaluation of commonly used cardiovascular drugs in inhibiting vonoprazan metabolism *in vitro* and *in vivo*

**DOI:** 10.3389/fphar.2022.909168

**Published:** 2022-08-16

**Authors:** Yiran Wang, Jihua Shi, Dapeng Dai, Jianping Cai, Shuanghu Wang, Yun Hong, Shan Zhou, Fangling Zhao, Quan Zhou, Peiwu Geng, Yunfang Zhou, Xue Xu, Qingfeng Luo

**Affiliations:** ^1^ Department of Gastroenterology, Beijing Hospital, National Center of Gerontology, Institute of Geriatric Medicine, Chinese Academy of Medical Sciences, Beijing, China; ^2^ Peking University Fifth School of Clinical Medicine, Beijing, China; ^3^ The Key Laboratory of Geriatrics, Beijing Institute of Geriatrics, Institute of Geriatric Medicine, Chinese Academy of Medical Sciences, Beijing Hospital/National Center of Gerontology of National Health Commission, Beijing, China; ^4^ Laboratory of Clinical Pharmacy, The Sixth Affiliated Hospital of Wenzhou Medical University, The People’s Hospital of Lishui, Lishui, China

**Keywords:** vonoprazan metabolism, cardiovascular drugs, drug–drug interactions, cytochrome P450, drug metabolism

## Abstract

As a novel acid-suppressing drug, vonoprazan shows the potential to replace traditional proton-pump inhibitors. With its widespread use, some adverse effects that require further study have emerged due to drug–drug interactions. Our study is the first experiment that evaluated the drug–drug interactions of eleven common cardiovascular drugs that inhibit vonoprazan metabolism *in vitro* and *in vivo*. Rat liver microsome incubation and molecular simulation docking were applied to explore the inhibition mechanism. Amlodipine and nifedipine showed inhibitory effects on vonoprazan metabolism in both rat and human liver microsomes in the first evaluation part *in vitro*. The inhibition mechanism analysis results demonstrated that amlodipine and nifedipine might inhibit the metabolism of vonoprazan by a mixed type of competitive and non-competitive inhibition. However, the pharmacokinetic data of the vonoprazan prototype revealed that amlodipine affected vonoprazan *in vivo* while nifedipine did not. Thus, more attention should be paid when amlodipine is prescribed with vonoprazan. Furthermore, the changes in its carboxylic acid metabolites MI hinted at a complex situation. Molecular simulation suggested the CYP2B6 enzyme may contribute more to this than CYP3A4, and further inhibitory experiments preliminarily verified this speculation. In conclusion, the use of vonoprazan with cardiovascular drugs, especially amlodipine, should receive particular attention in clinical prescriptions.

## 1 Introduction

Drug–drug interactions (DDIs) are one of the main reasons for adverse drug reactions but can be prevented with a comprehensive understanding (Subramanian et al., 2018). Study of the drug combinations that can cause DDIs and related mechanisms will assist doctors in making a more suitable choice when prescribing. Vonoprazan is a novel acid-suppressing drug activating as a potassium-competitive acid blocker (P-CAB). It shows some potential clinical advantages over traditional acid-related drug proton-pump inhibitors (PPIs) (Fallone et al., 2019) for a better pharmacological profile: rapid onset of action, long-lasting acid suppression, and control of nocturnal acidity (Savarino et al., 2021). The post-gastric endoscopic submucosal dissection (ESD) bleeding data revealed that vonoprazan has a lower onset rate than traditional PPIs (Shiratori et al., 2022). Regarding the treatment of *Helicobacter pylori* infection, the vonoprazan-involved triple regimen exhibits a much higher eradication rate (>90%) than empiric therapy (Rokkas et al., 2021). Even for some types of peptic ulcers, vonoprazan is recommended over PPIs in clinical practice guidelines (Kamada et al., 2021).

**FIGURE 1:**
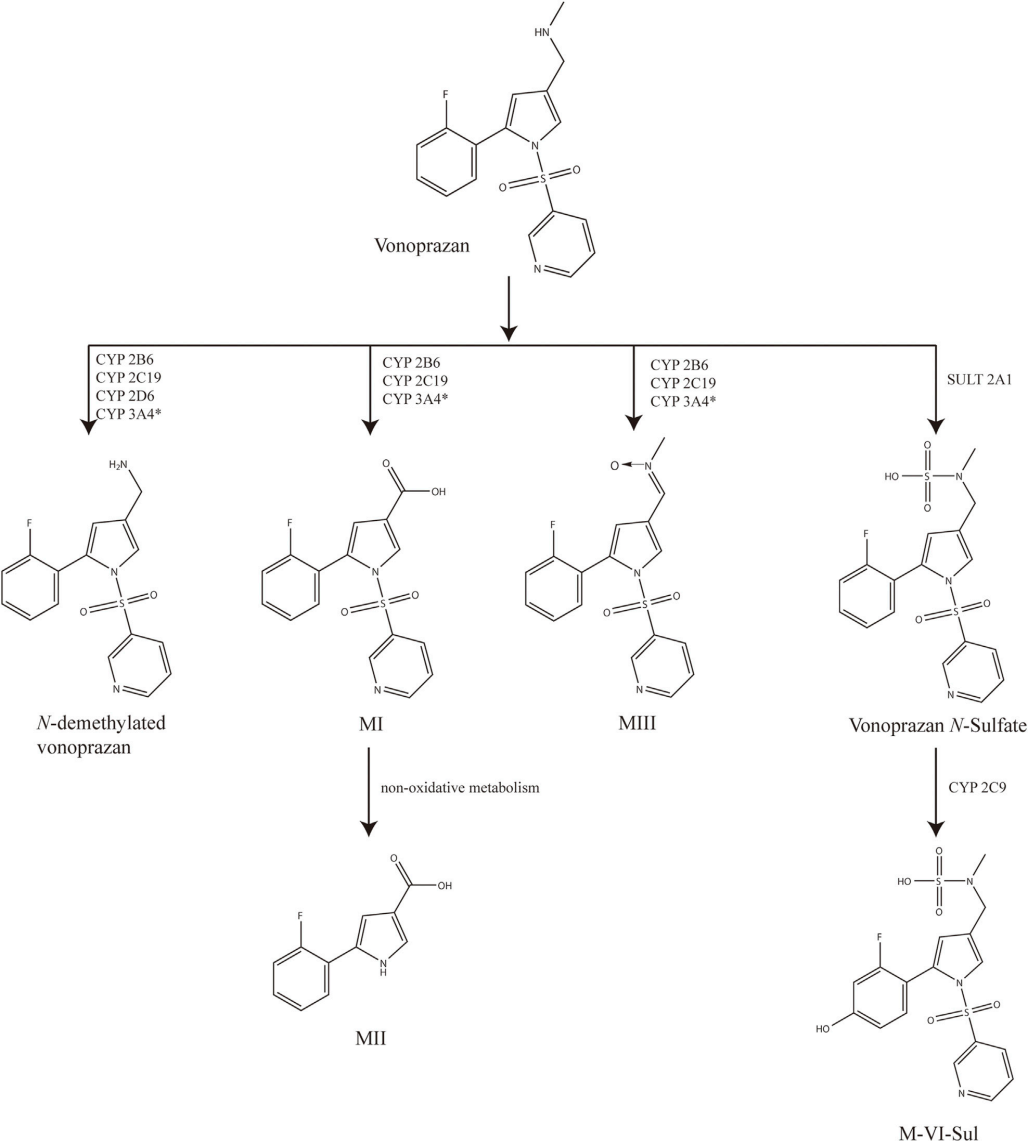
Postulated metabolic pathways of vonoprazan.

Vonoprazan is metabolized by multiple liver cytochrome P450 enzymes (CYP450) by oxidation (CYP3A4, CYP2B6, CYP2C19, and CYP2D6) and by sulfotransferase (SULT2A1) non-oxidatively. Its conversion into 5-(2-fluorophenyl)-1-(pyridin-3-ylsulfonyl)-1H-pyrrole-3-carboxylic acid (MI) is the main metabolic pathway (Figure 1), where the CYP3A4 was thought to play the leading role. Some factors that affect the activity of related enzymes (genotype or drug combination) may inhibit the metabolism of the drug and increase its blood concentration, leading to a higher probability of side effects. It is reported that the drug exposure of vonoprazan was not affected easily (Mulford et al., 2021), and vonoprazan seemed less likely to be involved in DDI situations than PPIs (Kato et al., 2021), which is probably due to the diversity of enzymes involved in metabolism. However, along with its widespread usage, side effects have emerged. The most common adverse effect of vonoprazan is stardust gastric mucosa (Yoshizaki et al., 2021). In addition, the onset of hypomagnesemia (Okamoto et al., 2021), hypergastrinemia (Kojima et al., 2018; Suzuki et al., 2021), *Clostridium difficile* infection (Watanabe et al., 2021), and hemorrhagic enterocolitis (Kambara et al., 2020) are also reported to be positively associated with the administration of vonoprazan. So, lower doses (Suzuki et al., 2018) and other ways to reduce the adverse effects are being studied. Furthermore, the exposure to vonoprazan could be increased in the triple regimen in the clinic (Sakurai et al., 2016). As for experimental research, Shen et al. (2020) explored whether the metabolism of vonoprazan could be inhibited by voriconazole, regardless of single or multiple doses. Hence, the stability of vonoprazan metabolism may have been overestimated previously, and more related studies are needed for rational drug use.

DDIs are more common in older people due to comorbidities. Cardiovascular drugs are among the most commonly used types of drugs, and they are also commonly referred to when studying DDIs in the clinic (Gallo et al., 2019). In older people, gastrointestinal-related side effects frequently appear when taking cardiovascular medicine. In addition, comorbidity with cardiovascular and digestive diseases is not rare. Guisado-Clavero et al. (2019) analyzed the medication patterns in 164,513 older adults in Barcelona (Spain). They found that acid-suppressing drugs (PPIs) were the top three most-used drugs in the cardiovascular system pattern. Therefore, as an acid-suppressing drug with great promise, vonoprazan is likely to be coadministered with cardiovascular drugs in clinical settings. To better understand the potential interactions between vonoprazan and cardiovascular drugs, we chose eleven common-used cardiovascular drugs to examine the inhibition effects of cardiovascular drugs on the vonoprazan metabolism both *in vitro* and *in vivo*. The CYP enzymes that those drugs inhibited are listed in Table 1. We used rat liver microsomes (RLMs), human liver microsomes (HLMs), rat models, and molecular simulation to evaluate the potential DDIs.

**TABLE 1:** The enzymes that eleven commonly used cardiovascular drugs mainly inhibited.

Drugs	Inhibited enzymes	References
Verapamil	CYP3A4	PMID:15689501
Diltiazem	CYP3A4	PMID: 11560871
Nifedipine	CYP3A4	PMID: 24399740
Amlodipine	CYP2B6, CYP2D6, CYP2C9, and CYP3A4	PMID: 10805063, 26721703
Amiodarone	CYP1A2, CYP2C9, and CYP2D6, CYP2J2, and CYP3A4	Nexterone FDA label, PMID: 26972388
Propafenone	CYP1A2 and CYP2D6	PMID: 17164694, 10945315
Quinidine	CYP2D6	PMID: 11061580
Irbesartan	CYP2C9 and CYP2J2	PMID: 10877007, 26632190
Valsartan	None	
Benazepril	None	
Captopril	None	

No literature support was obtained.

## 2 Materials and methods

### 2.1 Drugs and reagents

The cardiovascular drugs verapamil hydrochloride, diltiazem hydrochloride, nifedipine, amlodipine besylate, amiodarone hydrochloride, quinidine, propafenone, valsartan, irbesartan, benazepril hydrochloride, and captopril were all purchased from Aladdin (Shanghai, China). The vonoprazan was obtained from Shanghai Send Pharm, and its carboxylic acid metabolite, MI, was provided by Wuxi Apptec Co., Ltd. (Shanghai, China).

Diazepam from J&K Scientific Ltd. (Beijing, China) was selected as the internal standard (IS). Nicotinamide adenine dinucleotide phosphate (NADPH) was purchased from Coolaber (Beijing, China). Methanol and acetonitrile of high-performance liquid chromatography (HPLC) grade were obtained from Merck Company (Darmstadt, Germany). Formic acid was purchased from Sigma-Aldrich (St. Louis, United States). Ultra-pure water was produced by the reagent-standard water purification system Milli-Q (Millipore, United States). The RLMs were prepared according to our previously reported method (Wang et al., 2020), and the HLMs were purchased from Bioruler (Wuhan, China).

### 2.2 Animals and treatment

The Experimental Animal Research Center of Wenzhou Medical University provided specific pathogen-free (SPF) Sprague-Dawley rats (male, 350 ± 20 g, 3 months old). This research was conducted following the principles of the Basel Declaration. The protocol was approved by the Experimental Animal Management Committee of Wenzhou Medical University (grant number: xwydw 2019–650). The rats were reared in an environment with a temperature of 25 °C, relative humidity of 50 ± 10%, and light for 12 h, with uninterrupted water and a daily feed supply. After one week of rearing in the new environment and fasting overnight, the drug could be gavaged.

### 2.3 Instruments and experimental conditions

Acquity UPLC system (Waters Corp., Milford, United States) was used for the chromatographic separation. The liquid phase condition was composed of (A) pure acetonitrile and (B) 0.1% formic acid water, with a 0.4 ml/min flow rate in the mobile phase. An Acquity BEH C18 column (2.1 mm × 50 mm, 1.7 mM) at 40°C was applied. Each sample was injected with 2 μL, and every detection lasted for three minutes. The liquid phase gradient was set as follows: 0–0.5 min, 10–30% A; 0.5–1 min, 30–90%; 1–2 min, 90% A; 2–3 min, 90–10% A.

**FIGURE 2:**
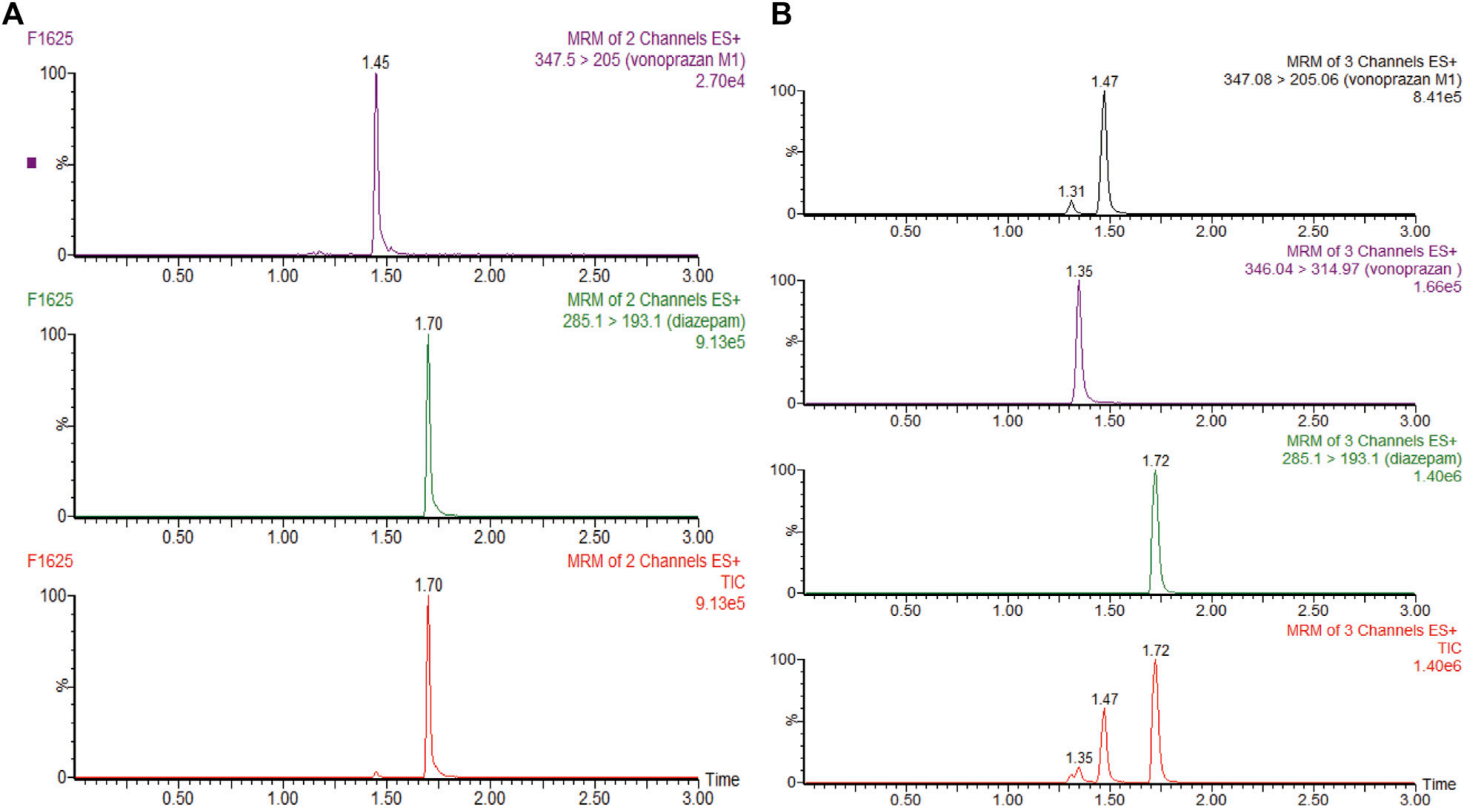
**(A)** UPLC-MS/MS chromatograms of vonoprazan metabolite MI and internal standard (IS) diazepam after incubation in rat liver microsomes or human liver microsomes. **(B)** UPLC-MS/MS chromatograms of vonoprazan, MI, and IS in a blood sample.

A positive-ion electrospray ionization (ESI) spectrum in the multiple reactive ion monitoring (MRM) mode was used for the ion quantification on a XEVO TQD triple quadrupole mass spectrometer. Detailed parameter settings for detecting diazepam (IS), vonoprazan, and its carboxylic acid metabolite MI are addressed in Shen et al. (2020). Data were collected and analyzed by Mass-Lynx 4.1 software (Waters Corp., Milford, United States), and typical UPLC-MS/MS chromatograms of liver microsomes (same in RLMs and HLMs) and plasma are shown in Figure 2 and Supplementary Figure S1.

### 2.4 Inhibition effect evaluation *in vitro*

**FIGURE 3:**
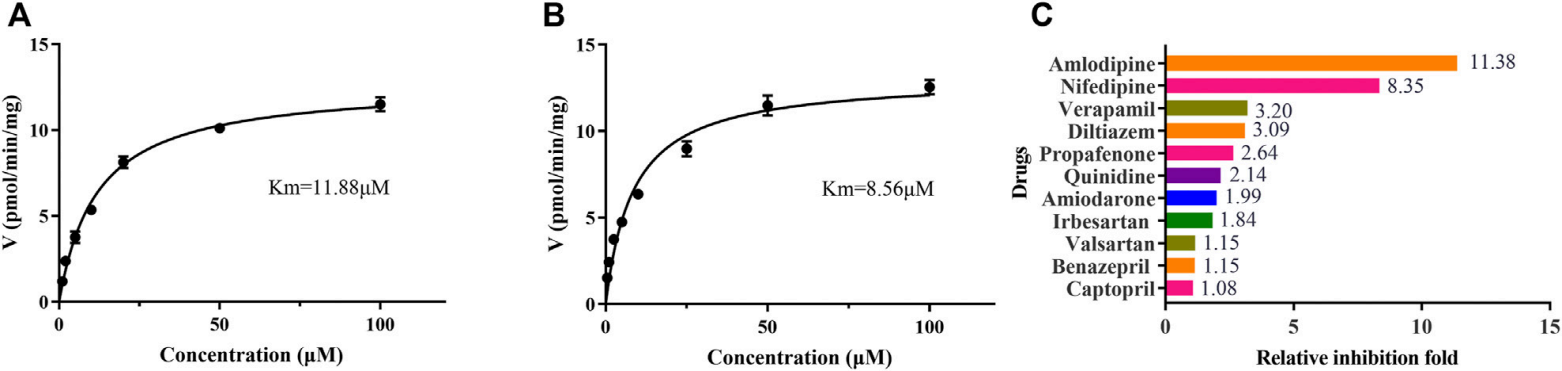
The Michaelis–Menten kinetics of vonoprazan in RLMs **(A)** and HLMs **(B)**, and the relative inhibition effects of eleven cardiovascular drugs exerted on vonoprazan **(C)**.

Eleven common-used cardiovascular drugs were chosen to detect their possible inhibition effect on vonoprazan. Each sample was prepared to have a roughly 100 µM concentration of the drug in the final mixture. The basic incubation system contained one drug (methyl alcohol as negative control), 10 µM vonoprazan (according to its detected Km, Figures 3A,B), RLMs (0.56 mg protein/ml), 100 mM potassium phosphate buffer (pH 7.4, with a final concentration of 0.1 mM), and deionized water. After preincubation at 37°C for 5 min in a shaking water bath, 20 mM NADPH was added to initiate the reaction, which lasted 30 min and was stopped with 200 ml of acetonitrile. The mixture was vortexed after adding 20 µL of diazepam and then centrifugated at 13,000 rpm for 5 min. A 2 µL aliquot of the final supernatant was injected into the UPLC–MS/MS system for analysis. The relative inhibition was calculated according to the relative MI decrease compared to the control (vonoprazan naturally metabolized in RLMs). Next, the drugs with more than a five-fold relative inhibition effect (amlodipine and nifedipine) were selected to determine the half maximal inhibitory concentration (IC_50_) in both RLMs and HLMs. According to the Km, inhibitors were set as eight concentration gradients (0, 1, 2.5, 5, 10, 25, 50, and 100 µM in the mixture) in a similar incubation condition (HLMs were used in 0.2 mg protein/ml). The same procedures were performed. Then, an inhibition mechanism experiment was conducted for further exploration. Four concentrations according to the IC_50_ of amlodipine and nifedipine were chosen to match 2.5, 5, 10, and 20 µM of vonoprazan, respectively. Both inhibitor and substrate were added to the incubation system described above. Every concentration match had three parallels. The MI concentration was detected using the same conditions to depict the Lineweaver–Burk and Dixon plots.

To preliminarily confirm the simulation results, we performed the inhibitory experiments on CYP2B6 *in vitro* (RLMs and HLMs). Ticlopidine and clopidogrel, classical CYP2B6 inhibitors (Nishiya et al., 2009; Zhang et al., 2011), were selected to verify that vonoprazan metabolism can be inhibited *via* CYP2B6. Meanwhile, the CYP2B6 probe drug bupropion was applied to ensure that amlodipine can influence the activity of the CYP2B6 enzyme.

### 2.5 Inhibition effect evaluation *in vivo*

Rats were randomly divided into three groups (*n* = 6). The amlodipine and nifedipine groups were administrated 10 mg/kg of amlodipine and nifedipine (dissolved in 0.5% CMC-Na), respectively, while the control group was treated with the same volume of CMC-Na. After 7 days, on the seventh day, all groups had gavage at a single dosage of 2 mg/kg vonoprazan.

Tail vein blood was collected into 1.5 ml centrifuge tubes with heparin sodium at different times after gavage (0.083, 0.25, 0.5, 1, 2, 3, 4, 6, 8, 12, and 24 h). Centrifugation at 4,000 rpm for 10 min was performed to obtain 100 µL of plasma, to which was added 200 µL of acetonitrile and 20 µL of diazepam IS. After vortex mixing and centrifugation at 13,000 rpm for 5 min, 5 µL of supernatant was obtained for analysis by UPLC-MS/MS.

### 2.6 Statistical analysis

The concentrations of vonoprazan and MI that were detected by UPLC-MS/MS were applied to GraphPad 7.0 (GraphPad Software Inc., United States) to calculate IC_50_ and depict plasma concentration-time curves and Lineweaver–Burk and Dixon plots. The pharmacokinetic parameters of the compartment analysis were calculated using DAS (version 3.2.8; The People’s Hospital of Lishui) and expressed as mean ± standard deviation. The t-test was carried out by SPSS 25.0 (SPSS Inc., United States) to compare those parameters. *p* value < 0.05 was considered statistically significant.

### 2.7 Molecular docking

Molecular docking was performed according to the methods previously reported (Sevrioukova and Poulos, 2017) to explore the possible inhibition mechanism of these drugs. In detail, the crystal structures of CYP2B6, CYP2C9, CYP2D6, and CYP3A4 were downloaded from the RCSB PDB database (https://www.rcsb.org/), and the molecular structures of vonoprazan, amlodipine, and verapamil were obtained from Pubchem in NCBI (https://pubchem.ncbi.nlm.nih.gov/). Then, Pymol (Version 2.5.2, Schrödinger, United States) was used for the structure optimization. AutoDock Vina (Version 1.2.0, The Scripps Research Institute, United States) was used for the molecular docking and affinity value calculation with the default parameters. The predicted docking structures with the highest affinity value were chosen for further simulation, and the docking results were then visualized by Pymol software.

## 3 Results

### 3.1 Inhibition effects of cardiovascular drugs on vonoprazan metabolism *in vitro*

**FIGURE 4:**
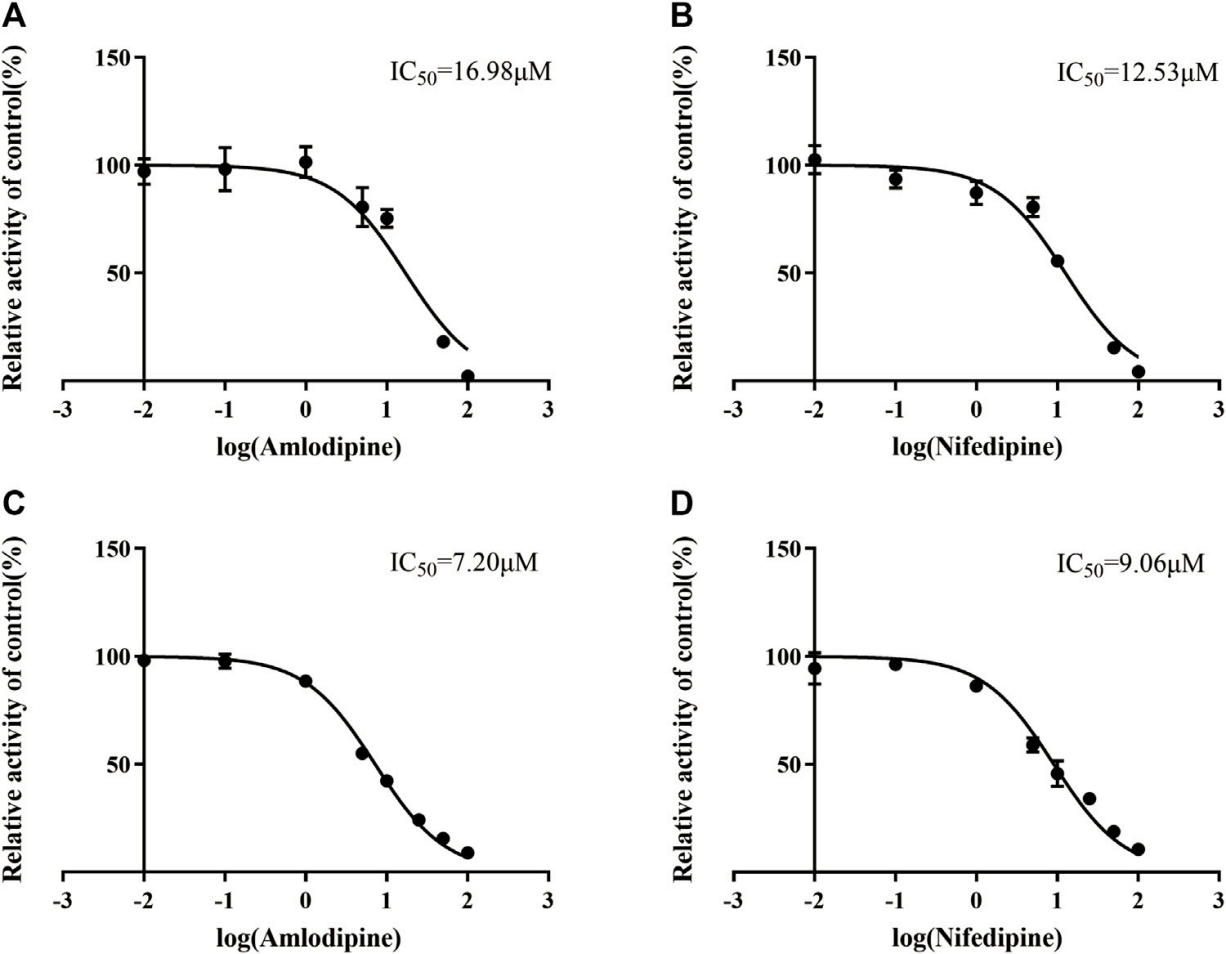
The IC_50_ values in RLMs inhibitory effect of amlodipine **(A)** and nifedipine **(B)**. The IC_50_ values in HLMs inhibitory effect of amlodipine **(C)** and nifedipine **(D)**.

**FIGURE 5:**
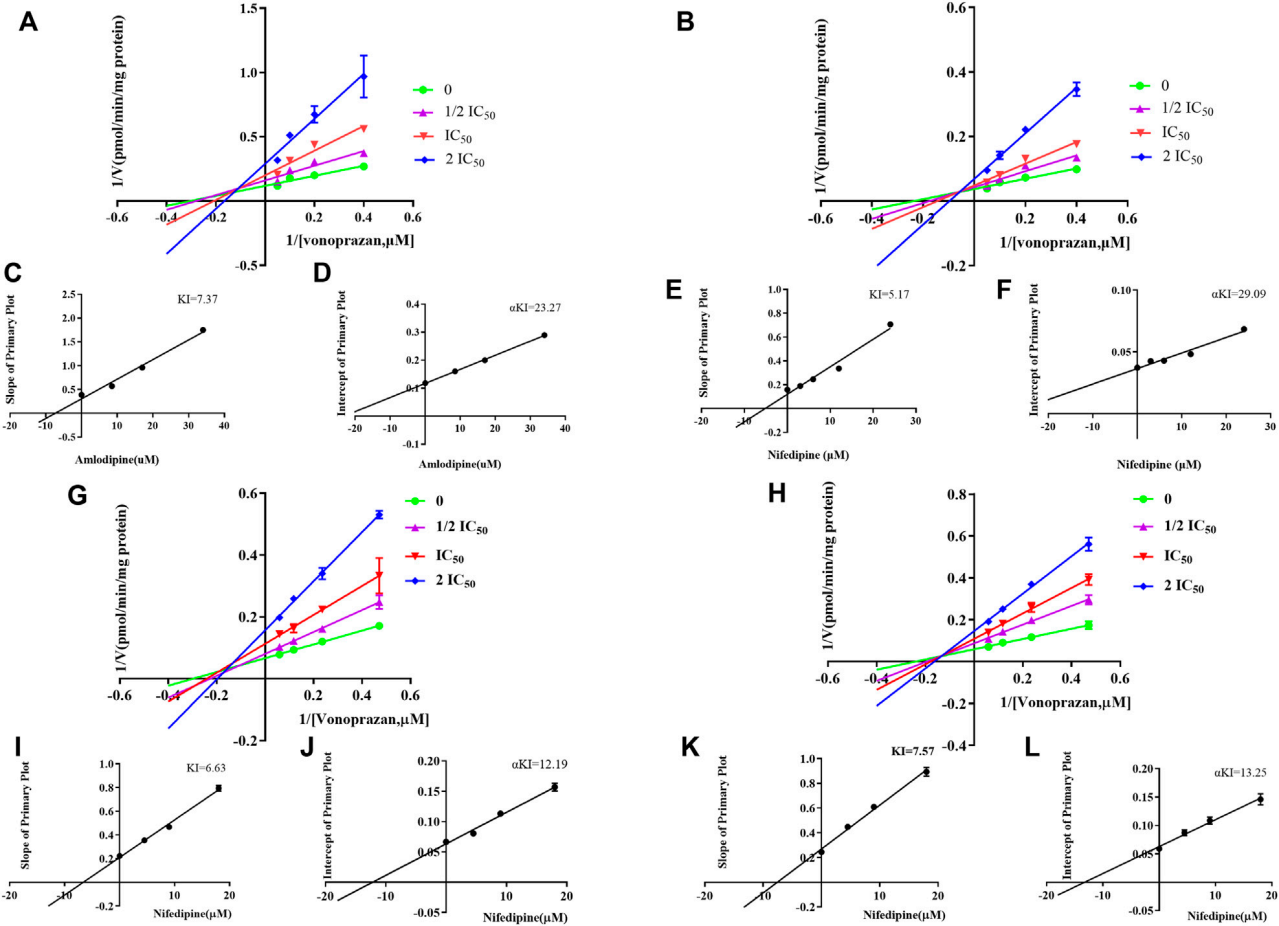
The Lineweaver–Burk and Dixon plots of amlodipine and nifedipine inhibition on vonoprazan in RLMs: **(A)** Lineweaver–Burk plot of amlodipine. **(B)** Lineweaver–Burk plot of nifedipine **(C)** Slope of the primary plot of amlodipine **(D)** Intercept of the primary plot of amlodipine. **(E)** Slope of the primary plot of nifedipine. **(F)** Intercept of the primary plot of nifedipine. The Lineweaver–Burk and Dixon plots of amlodipine and nifedipine inhibition on vonoprazan in HLMs: **(G)** Lineweaver–Burk plot of amlodipine. **(H)** Lineweaver–Burk plot of nifedipine. **(I)** Slope of the primary plot of amlodipine. **(J)** Intercept of the primary plot of amlodipine. **(K)** Slope of the primary plot of nifedipine. **(L)** Intercept of the primary plot of nifedipine.

**FIGURE 6:**
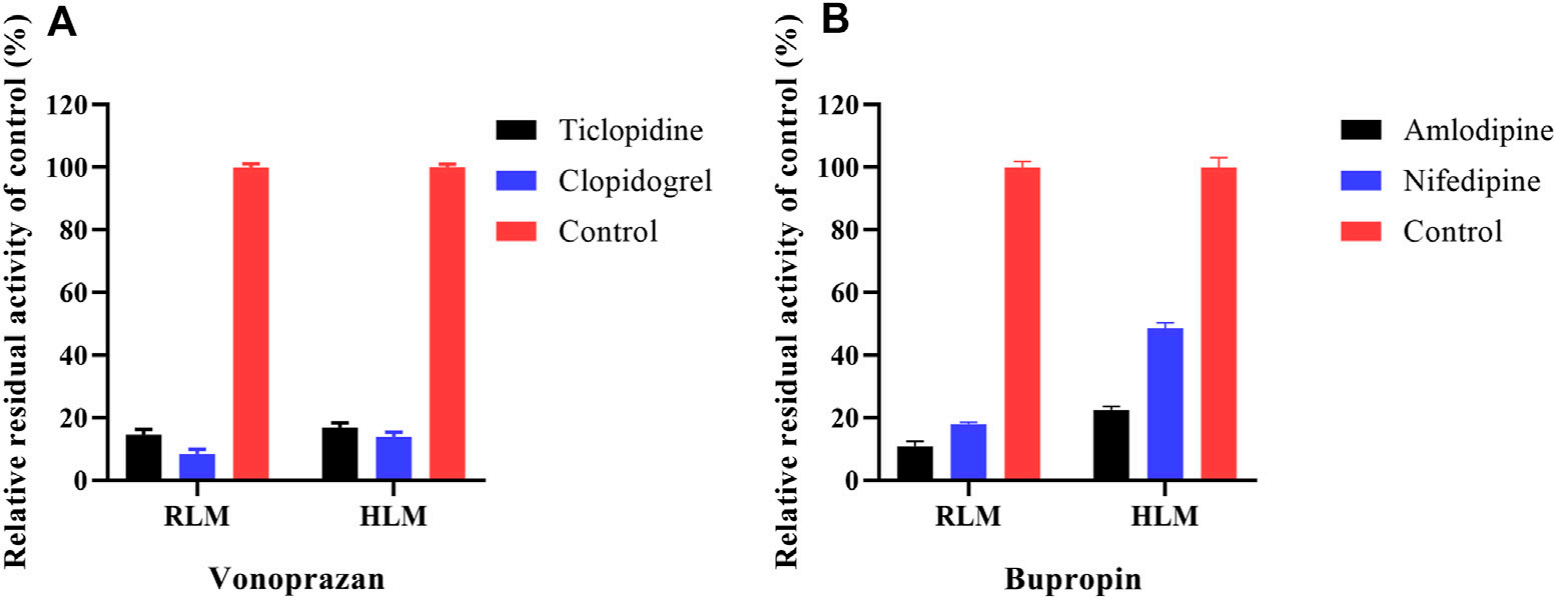
The inhibition of vonoprazan by ticlopidine and clopidogrel with relative residual activity of control **(A)**. The inhibition of bupropion by amlodipine and nifedipine with relative residual activity of control **(B)**.

To identify cardiovascular drugs that may have interactions with vonoprazan, eleven clinical commonly used prescription drugs were screened with rat liver microsome. Amlodipine and nifedipine exhibited strong inhibition effects on vonoprazan metabolism, as shown in Figure 3C (more than 5-fold). The IC_50_ values of amlodipine and nifedipine in RLMs and HLMs are shown in Figure 4. The Lineweaver–Burk and Dixon plots (Figure 5) revealed the inhibition constant and mechanism. These data indicated these two drugs inhibited the metabolism of vonoprazan both competitively and noncompetitively. The inhibition of vonoprazan by ticlopidine and clopidogrel with relative residual activity of control is shown in Figure 6A. In addition, the inhibition of bupropion by amlodipine and nifedipine with relative residual activity of control is shown in Figure 6B. The inhibitory experiments regarding CYP2B6 verified that vonoprazan could be affected *via* CYP2B6. In addition, amlodipine influenced CYP2B6 more than nifedipine, rather than only inhibiting CYP3A4 as once believed.

### 3.2 Inhibition effects of amlodipine on vonoprazan metabolism *in vivo*

**FIGURE 7:**
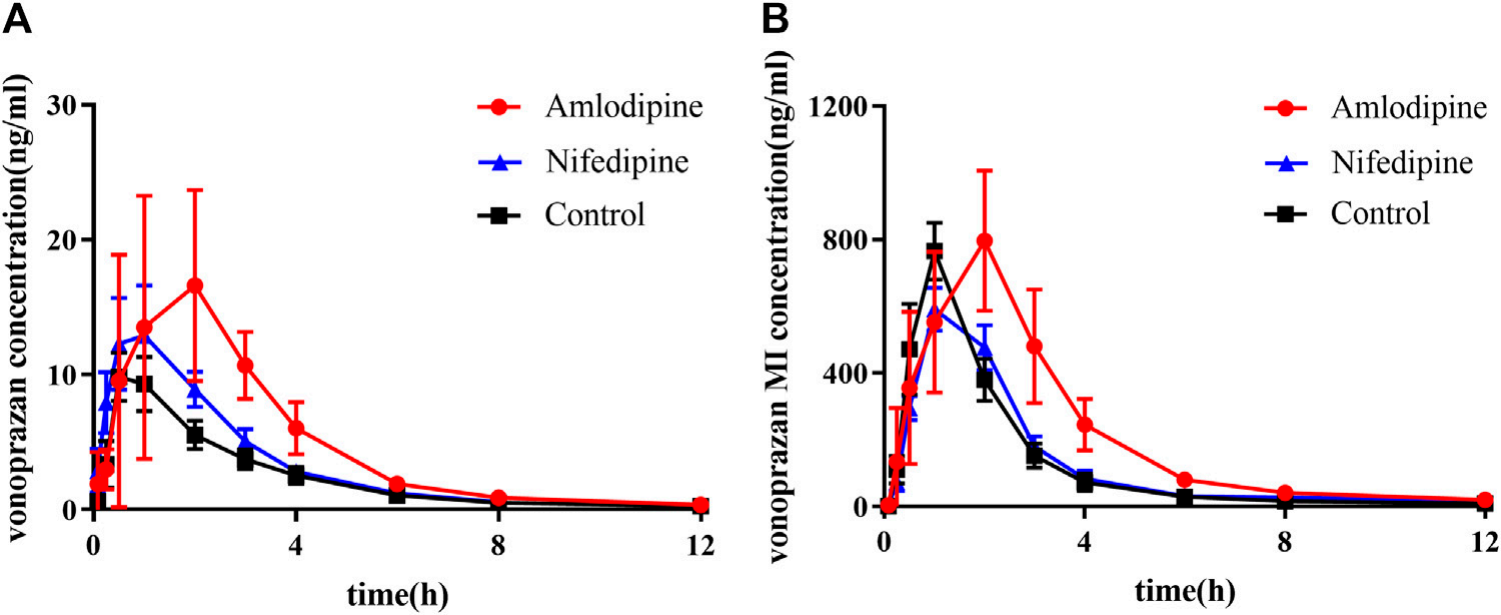
Mean plasma concentration–time curves of vonoprazan prototype and metabolite MI in amlodipine, nifedipine, and control groups. **(A)** Mean plasma concentration–time curves of vonoprazan prototype in control and amlodipine groups. **(B)** Mean plasma concentration–time curves of vonoprazan metabolite MI in control and amlodipine groups (*n* = 6).

The mean plasma concentration-time curves of vonoprazan and its metabolite MI are shown in Figure 7, and their detailed pharmacokinetic parameters are detailed in Table 2. For the amlodipine-pretreated group, the values of the area under the curve (AUC), the time of peak concentration (Tmax), and the peak concentration (Cmax) of vonoprazan increased nearly twice as much as those of the control group. In contrast, the values of apparent volume of distribution (Vz/F) and clearance (CLz/F) decreased approximately by half compared with those of the untreated control group. Regarding the carboxylic acid metabolite MI, the values of AUC, mean residence time (MRT) (0-t), and MRT (0-∞) increased 1.6-fold, 1.4-fold, and 1.2-fold, respectively. However, the values of Vz/F and CLz/F fell by 57 and 38%, respectively. Although nifedipine showed slightly stronger inhibition ability *in vitro*, the *in vivo* results did not validate this stronger inhibition.

**TABLE 2:** Pharmacokinetic parameters of vonoprazan and MI (mean ± SD, *n* = 6).

Parameters	Unit	Vonoprazan		MI	
		Control group	Amlodipine group	Control group	Amlodipine group
AUC_(0-t)_	ug/L*h	28.78 ± 5.14	58.45 ± 21.11**	1540.00 ± 174.96	2548.38 ± 459.43**
AUC_(0-∞)_		29.14 ± 5.31	59.01 ± 20.86**	1572.36 ± 189.42	2584.16 ± 449.51**
MRT_(0-t)_	h	2.72 ± 0.09	2.93 ± 0.30	2.13 ± 0.14	2.92 ± 0.19**
MRT_(0-∞)_		2.87 ± 0.17	3.06 ± 0.38	2.44 ± 0.26	3.11 ± 0.31**
t_1/2_z		1.89 ± 0.37	1.73 ± 0.65	2.91 ± 1.51	1.91 ± 0.76
T_max_		0.75 ± 0.27	1.67 ± 0.52**	1 ± 0	1.58 ± 0.67
Vz/F	L/kg	190.50 ± 41.60	95.48 ± 51.30**	5.28 ± 2.50	2.25 ± 1.11*
CLz/F	L/h/kg	70.60 ± 13.19	36.82 ± 10.08**	1.29 ± 0.16	0.79 ± 0.14**
C_max_	ug/L	10.23 ± 1.88	18.99 ± 8.30*	765.65 ± 85.10	835.77 ± 175.74

*p < 0.05 indicates the statistical difference between the two groups. **p < 0.01 indicates the statistical difference between the two groups.

AUC, the area under the concentration–time curve; MRT, the mean residence time; t1/2, half-life period; Tmax, maximum plasma time; Vz/E, apparent volume of distribution; CLz/F, clearance; Cmax, peak plasma concentration.

### 3.3 Molecular docking prediction of amlodipine and vonoprazan

**FIGURE 8:**
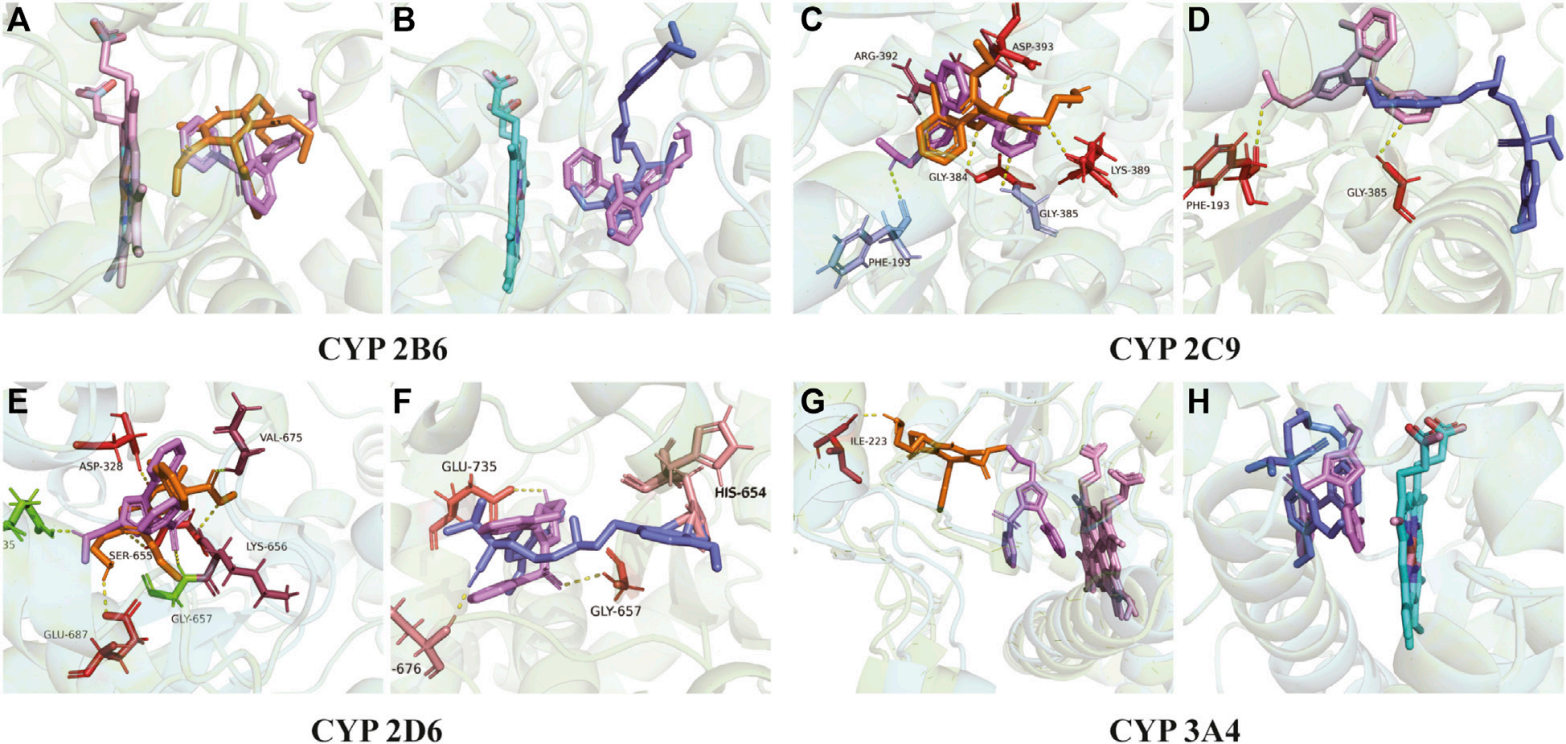
Molecular docking. **(A)** Action position of amlodipine and vonoprazan in CYP 2B6 enzyme. **(B)** Action position of verapamil and vonoprazan in CYP 2B6 enzyme. **(C)** Action position of amlodipine and vonoprazan in CYP 2C9 enzyme. **(D)** Action position of verapamil and vonoprazan in CYP 2C9 enzyme. **(E)** Action position of amlodipine and vonoprazan in CYP 2D6 enzyme. **(F)** Action position of verapamil and vonoprazan in CYP 2D6 enzyme. **(G)** Action position of amlodipine and vonoprazan in CYP 3A4 enzyme. **(H)** Action position of verapamil and vonoprazan in CYP 3A4 enzyme. The yellow dotted lines connect the acting sites between drugs and enzymes *via* hydrogen bonding.

Figure 8 exhibits the overlap of vonoprazan and amlodipine and verapamil, two typical drugs with diverse inhibition effects, in the mimetic enzyme models. The positions of three drugs in each enzyme are shown in Supplementary Figure S2. The larger contact area implied a stronger direct DDI, consistent with a higher affinity value. It can be observed that verapamil combined with vonoprazan more tightly in CYP3A4, which is in accord with the fact that verapamil is a stronger CYP3A4 inhibitor than amlodipine. Those pictures also depict the assumed acting sites through which the inhibitors affect enzymes, providing indirect evidence. Compared to verapamil, amlodipine had more assumed acting sites in CYP2C9, CYP2D6, and CYP3A4, though verapamil displayed higher affinity values (Table 3). Meanwhile, amlodipine has a larger contact area with vonoprazan in CYP2B6, suggesting that this enzyme may play a more important role in the studied DDIs.

**TABLE 3:** The affinity of amlodipine and verapamil with CYP450 enzymes (mean ± SD, *n* = 9).

	Amlodipine	Verapamil	*F* value	*p* value
CYP 2B6	2.678 ± 0.540	1.022 ± 0.642	0.231	0.00
CYP 2C9	4.444 ± 0.855	4.167 ± 0.469	3.702	0.405
CYP 2D6	7.3 ± 0.397	8.3 ± 0.371	0.234	0.00
CYP 3A4	6.833 ± 0.25	7.878 ± 0.179	0.634	0.00

## 4 Discussion

As a member of the novel group of acid-suppression drugs, P-CAB, the use of vonoprazan spreads quickly. Although numerous clinical pharmacology studies and clinical trials of vonoprazan are underway (Abdel-Aziz et al., 2021), few pay much attention to the DDIs of vonoprazan. Our previous study (Wang et al., 2020) focused on how vonoprazan affected other CYP450 enzymes. By applying drug probe cocktails and LC/MS, we assumed vonoprazan inhibited four CYP enzymes: CYP2B6, CYP2C9, CYP2D6, and CYP3A4. Considering that vonoprazan itself is also mainly metabolized by those four enzymes (Kogame et al., 2017), we suspected its metabolism might be interfered with by CYP450 inhibitors or inducers, such as drugs for the treatment of mental/neurological diseases (carbamazepine), cardiovascular drugs (quinindium), or antifungal drugs (ketoconazole). Due to the widespread clinical use of cardiovascular drugs, they are likely to be co-administrated with vonoprazan. To date, no study can tell whether they would have DDIs, let alone explain the related mechanism.

In this study, we chose eleven commonly prescribed cardiovascular drugs for our first stage of DDI evaluation: verapamil, diltiazem, nifedipine, amlodipine, amiodarone, quinidine, propafenone, irbesartan, valsartan, benazepril and captopril (as a control drug not metabolized by liver enzymes). Unexpectedly, the preliminary *in vitro* evaluation results showed that amlodipine and nifedipine exhibited the highest inhibitory effect (with more than 5-fold) on vonoprazan, while other CYP3A4 inhibitors, verapamil, diltiazem and amiodarone only exhibited slightly increased inhibition effects, although they were reported to have the same (Zhou, 2008) or even stronger (Sychev et al., 2020) inhibition on CYP3A4 than that of amlodipine or nifedipine. Next, we evaluated the IC_50_ values of amlodipine and nifedipine that reconfirmed their inhibition effects on vonoprazan in both RLMs and HLMs, showing a stronger inhibition in HLMs. The inhibitory mechanism analysis results suggested that amlodipine and nifedipine inhibited the metabolism of vonoprazan by primarily competitive with some non-competitive inhibition. A similar inhibition mechanism has been reported for the effect that amlodipine exerts on CYP2J2 enzyme activity (a relatively high component of competitive inhibition) (Ikemura et al., 2019).

The *in vivo* pharmacokinetic data of the vonoprazan prototype differed from the *in vitro* results. A high inhibitory effect on vonoprazan metabolism was detected in rats when pretreated with amlodipine for two weeks. The AUC of vonoprazan increased nearly two times, which means that the drug concentration in blood was elevated and indicates that the dosage should be adjusted when coadministered in the clinic to avoid possible side effects. The prolonged Tmax implied that the metabolism of vonoprazan decreased under the inhibition. Considering that amlodipine is a long-acting antihypertensive drug, its slower absorption and release might lead to the continuity in inhibiting vonoprazan. It reminded us that doctors must be careful about dosing frequency in clinical practice. However, this inhibition was not observed in the nifedipine group. Thus, amlodipine may be more likely to affect vonoprazan in the clinic, while nifedipine may not. This is probably attributed to the better bioavailability of amlodipine than nifedipine (Stopher et al., 1988; Park and Choi, 2019), leading to a stronger inhibition *in vivo*. Amlodipine and nifedipine both belong to the category of dihydropyridine calcium-channel blockers. These drugs block the inward movement of calcium by binding to L-type calcium channels in the heart and arteriolar vasculature, leading to the relaxation of vascular smooth muscle and the dilation of arterioles (McKeever and Hamilton, 2019). As one of the most-prescribed drugs for patients with hypertension (Augustin et al., 2018), amlodipine has a better effect on controlling blood pressure than nifedipine (Huang et al., 2019). In addition, amlodipine is generally taken for a long period of time, while nifedipine is usually used temporarily or only for emergencies. Based on these facts, the combination of amlodipine and vonoprazan must be monitored carefully.

When considering only the metabolic path from vonoprazan to MI for the amlodipine group, the changes in the pharmacokinetic data of vonoprazan metabolite MI in rat blood did not decline as expected, even though the production of MI from vonoprazan was highly suppressed by amlodipine in the incubation system. The situation was not as simple as previous results of the DDIs related to vonoprazan (Shen et al., 2020): if metabolism is inhibited, the blood concentration of the substrate prototype will increase while that of the metabolite would decrease. The plasma level of MI I increased while the prototype increased, hinting that the other metabolic pathways may be impeded more.

CYP3A4 is often credited with responsibility for the DDIs between amlodipine and other drugs, but it is not sufficient to explain our results. Although amlodipine is mainly metabolized by CYP3A4 (Zhu et al., 2014), it also inhibits CYP3A4 and CYP2C9 (Krasulova et al., 2017). The multiple-CYP enzyme repression may contribute to this. Thus, we added the CYP2B6, CYP2C9, and CYP2D6 enzymes and verapamil (strong CYP3A4 inhibitor) into molecular simulation docking for further mechanism exploration. The larger interaction area with vonoprazan and stronger affinity with enzymes implied that amlodipine probably most impacted the vonoprazan through CYP2B6, which had not been noticed before. Our further step inhibitory experiments about CYP2B6 *in vitro* indicated that vonoprazan could be quite strongly inhibited, although CYP2B6 and amlodipine could suppress the enzyme activity of CYP2B6. This may serve as a clue to explain why amlodipine had the inhibition effect while verapamil did not, suggesting CYP2B6 plays a more important role in vonoprazan-involved DDIs and needs more attention in future studies.

Kong et al. (Kong et al., 2020) used a pharmacokinetics prediction model to demonstrate an extrahepatic metabolism of vonoprazan *in vivo*, hinting a considerable first-pass elimination. If amlodipine also inhibited this path, the slight rise of MI in blood could be explainable. This may partly explain the inconsistency between the *in vitro* and *in vivo* MI results. Other clinical studies have also shown that considering a certain CYP activity/genotype was not enough to predict the clinical outcome of vonoprazan in combination with other drugs (Sugimoto et al., 2020). Hence, further studies are needed for an explicit conclusion.

The limitation of this study is obvious. As a newly developed drug, the metabolic pathway of vonoprazan is not comprehensively clear, and it is hard to identify all the metabolic products. In this study, we only synthesized the major carboxylic acid metabolite MI, and we know nothing about the impacts of selected cardiovascular drugs on the production of the other three metabolites. As an acid-suppressing drug like PPIs (Manolis et al., 2020), the actual side effects of long-term and combined usage of vonoprazan still require deep clinical investigations. However, our *in vitro* and *in vivo* pharmacokinetic data on the interactions between cardiovascular drugs and vonoprazan can give some direction for analysis (Fuhrmann et al., 2019), thus reducing related research costs for drug interaction studies initiated by pure speculation (Kirigaya et al., 2020).

Taken together, our data indicate that some cardiovascular drugs, especially amlodipine, can inhibit the metabolism of vonoprazan. The actual situations and mechanisms are more complex than presumed, and the combination of these drugs should be monitored carefully in clinical prescriptions.

## Data Availability

The original contributions presented in the study are included in the article/Supplementary Materials. Further inquiries can be directed to the corresponding author.
