# Supplementary material for: Evaluation of commonly used cardiovascular drugs in inhibiting vonoprazan metabolism in vitro and in vivo
Source: Front Pharmacol. 2022 Aug 16;13:909168. doi: 10.3389/fphar.2022.909168 (PMC9424819; doi:10.3389/fphar.2022.909168)
Supplement: Supplementary file 1 [file DataSheet1.PDF]

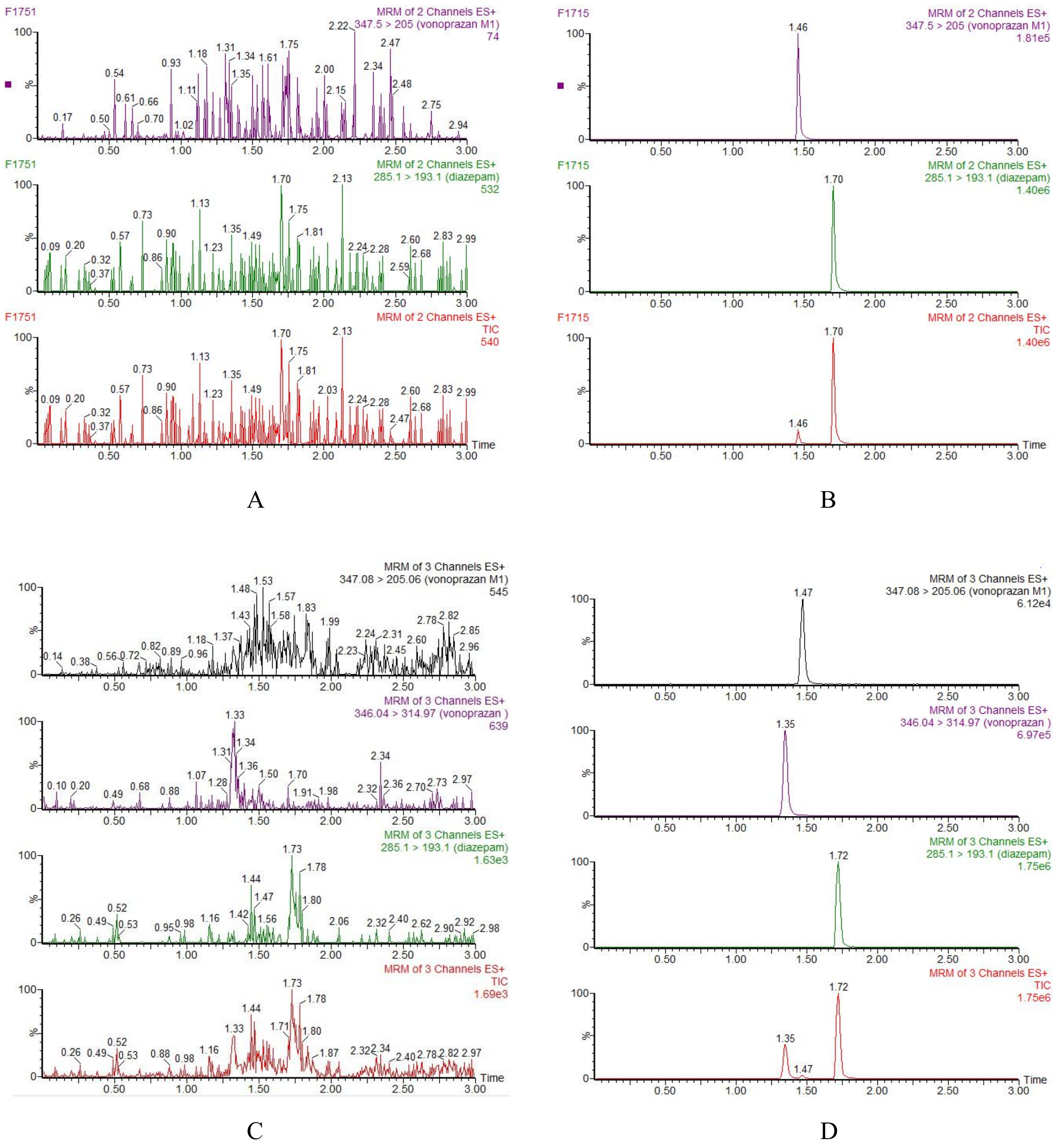

Supplemental Figure S1. UPLC-MS/MS chromatograms. (A) blank of rat liver microsome. (B) standered solution of MI. (C) blank of rat blood. (D) standered mixed solution of vonoprazan prototype and MI.

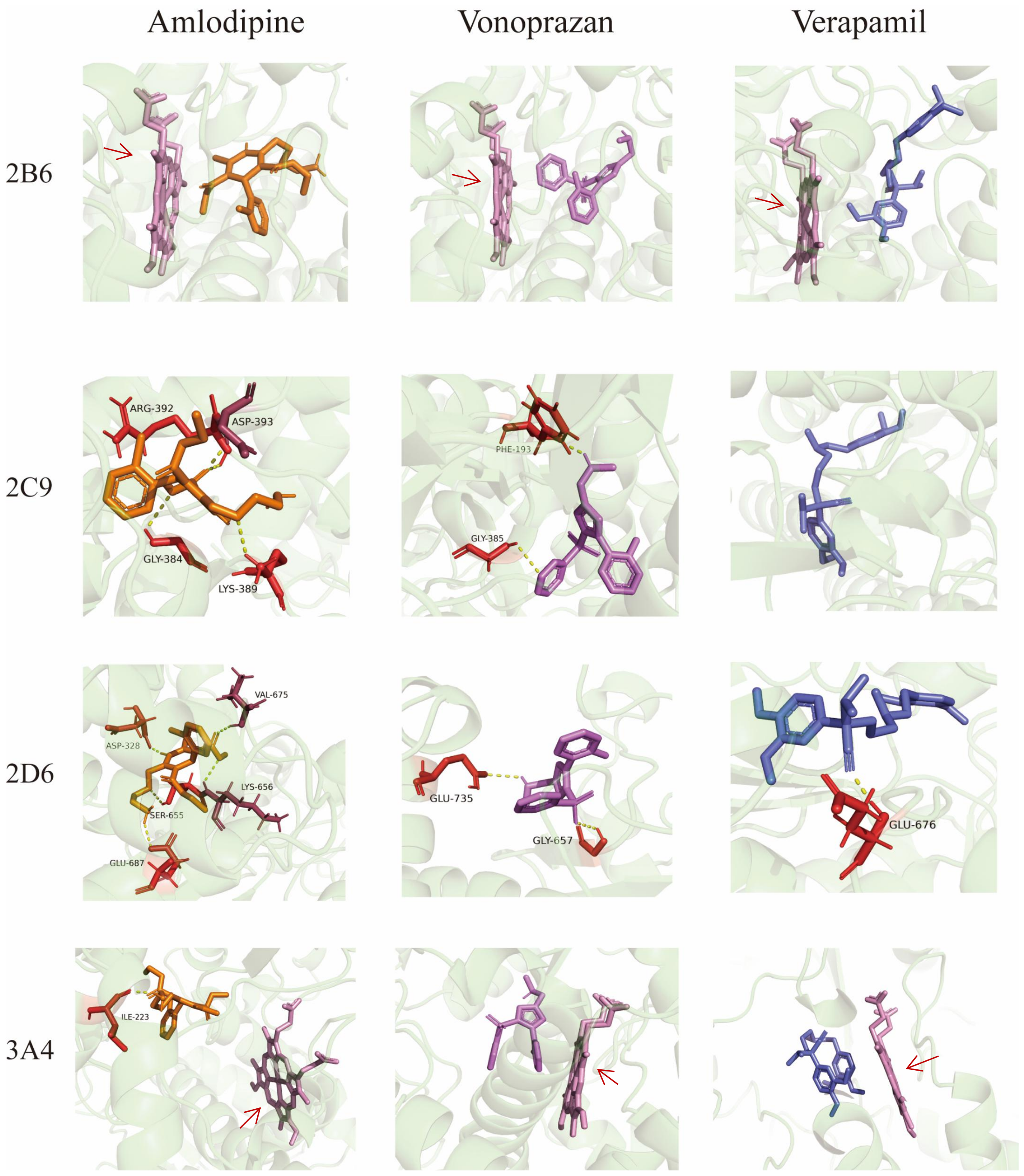

Supplemental Figure S2. The positions of three drugs in each enzyme. The yellow dotted lines connect the acting sites between drugs and enzymes. The substance pointed by red arrow is protoheme.
